# Shape-Dependent Strength of Al Si9Cu3FeZn Die-Cast Alloy in Impact Zone of Conformal Cooling Core

**DOI:** 10.3390/ma15155133

**Published:** 2022-07-24

**Authors:** Jarosław Piekło, Andriy Burbelko, Aldona Garbacz-Klempka

**Affiliations:** Faculty of Foundry Engineering, AGH University of Science and Technology, Al. A. Mickiewicza 30, 30-059 Kraków, Poland; jarekp60@agh.edu.pl (J.P.); abur@agh.edu.pl (A.B.)

**Keywords:** die casting, conformal cooling, conventional cooling, temperature analysis, strength analysis, microstructural analysis

## Abstract

This article presents the results of shape-dependent strength analyses in die-castings from traditional (straight-drilled) and conformal core-cooling moulds. Cores with a traditional cooling layout were made of H13 steel using machining, and the working sections of the conformal cores were made using the selective laser melting method (SLM). Two series of casts were produced in the same mould. For Series A, the mould was fitted with traditional cooling cores, and for Series B, the same mould was fitted with conformal ones. The cast specimens were subjected to two weeks of natural ageing. The strength testing of the casts determined the levels of the destructive forces. The destructive forces in the core-cooling impact zones were approximately 28% higher in the B samples than they were in the A samples. The impact of the alloy’s porosity, density, and microstructure on the strengths of the casts was demonstrated. The alloy densities in the central (broken-off) fragments of the casts from Series A were 2.6646 g/cm^3^; these were 2.6791 g/cm^3^ in the cases of casts from Series B. The values of the secondary dendrite arm space (SDAS) ranged from 6 to 13 μm in the analysed cross-section of the set of the A casts, and between 3 and 12 μm in the same zone of the set of the B casts. The results of the experimental determinations of the casts porosity levels and SDAS parameters were compared with the results of numerical simulations that were carried out in ProCAST software.

## 1. Introduction

Aluminium casts for the automotive industry are quite often produced by using high-pressure die-casting (HPDC) technology. The application of this technology and the optimum design of moulds yield products with very good dimensional accuracy, high strengths, and good surface smoothness. It is equally important to have the possibility of achieving low levels of alloy impurities and gas porosity, and high cast tightness [1]. Die casting is being increasingly applied in the production of structural aluminium castings, which are beginning to replace the pressed-steel components of automobile frames. Components that can be produced as a single element and cast from an Al-Si-based alloy would require a combination of several press-made sub-components if they were to be made of steel [2].

Guaranteeing the high quality of a cast and the durability of a die-casting mould requires an optimum design in the cooling system, which plays a very important role in controlling the temperature of the mould. A mould can be compared to a heat exchanger—part of the heat that is emitted by a solidifying alloy is evacuated by the thermal stabilization system’s liquid that circulates in the mould’s channels, and some directly dissipates into the environment through the surface of the mould. During the casting process, the thermal stabilization channels provide cooling for the most thermally loaded areas of the inserts and form cores of the casting mould.

A typical cooling system is composed of a working fluid temperature control system, a pump, hoses, supply and discharge lines, and cooling channels in the mould and cores. The intensive dissipation of heat from an alloy to the working fluid in the channels is proportional to the heat-transfer coefficients of the interfaces among a mould’s material, casting alloy, and working fluid as well as to the heat-transfer coefficient of the mould’s material. This also depends on the geometry of the cooling circuit, its outer surface area, and its distance from the surface of the mould cavity.

**Figure 1:**
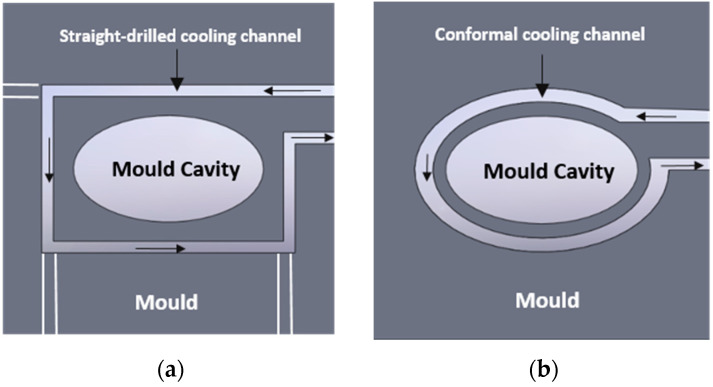
Schematics of the straight-drilled cooling channel (**a**) and conformal cooling channel (**b**) [3].

The optimum distribution of the cooling channels in a mould should eliminate or reduce the sizes of any hot spots in the cast, helping to ensure a proper solidification sequence. Accelerating the alloy solidification process in the hot spot areas results in reductions in the volume of any shrinking defects. Similarly, the single work cycle of a high-pressure machine (HPDC machine) is shortened. The traditional execution of the channels relies on drilling their particular sections in a mould block. These drilled channels consist of straight sections of a circular profile. For this reason, their distances from the surface of a mould cavity are not consistent; this leads to a non-optimal field of temperature distribution in the mould as well as the non-uniform cooling of the cast component. As a consequence, this may lead to the formation of cast porosities and the cast’s deformation. On the other hand, in the case of a conformal cooling system, the path of its channels can be adapted to the shape of the surface to be cooled—see Figure 1.

The aim of the research work was to determine the influence of the optimization of the pressure mould’s cooling method on the casting strength. In such a case, the increase in strength should result from a decrease in the share of shrinkage porosity in the casting and from the decrease in the dimensions of the microstructure cells as a result of the use of a conformal cooling system. The location and trajectory of the cooling channels depend on the shape of the casting and the construction of the mould; hence the obtained results are reliable only in relationship to the properties of the same type of castings that are made in a mould with a traditional cooling system. This comparison applies to those parts of the casting that are directly affected by the conformal cooling system. The tests were carried out on automotive castings that were produced by the HPDC technology in a two-cavity mould. Two series of castings were made in the same mould. Set A of the castings was produced with a set of traditional cores; then, a Set B was obtained after replacing the traditional cores with conformal ones.

The indirect goal of the work was also the design and fabrication of cores with a conformal cooling system using the SLM method, which can be used in the mass serial production of castings. Issues that are related to the differences in the form strength of the pressure castings that were caused by the increase in cooling efficiency through the use of a conformal cooling system are not presented in the literature.

**Figure 2:**
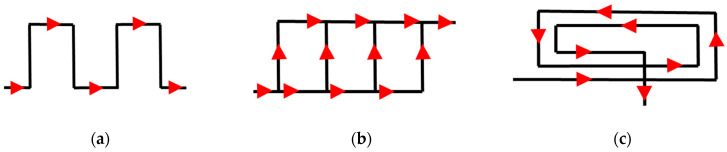
Diagram of three basic liquid flow patterns (2D example) in mould cooling channels: zig-zag (**a**); parallel (**b**); spiral (**c**).

Many authors have confirmed that a conformal layout of cooling channels leads to a more uniform temperature distribution in the cast and lowering of the average mould’s temperature [4,5]; this impacts an alloy’s microstructure and prevents the formation of shrinkage porosity [6,7]. Applying conformal cooling channels in the HPDC process improves the surface quality of casts by reducing the volume of the cooling liquid that is sprayed on the forming surfaces; this is possible thanks to the higher rate of mould cooling. In many cases, the conformal geometry of cooling channels eliminates 90° connections of the drilled channels. Such perpendicular connections cause sharp drops in cooling liquid pressure, and their elimination increases the efficiency of the cooling [8,9]. The rules for designing traditional cooling channels are well-known; they come down to determining each channel’s diameter, the distance of each channel’s surface from the surface of the mould cavity, and the distances between its adjacent channels. Designing channels in the parts of pressure die-cast and injection moulds that have been fabricated using the SLM method extends the range of the admissible design solutions as compared to the limitations that arise from machining methods. In such cases, a constructor may focus on improving the efficiency and functionality of a mould cooling system more than he/she has before. The design solutions of conformal cooling systems that are presented in the literature are mostly related to injection moulds. Following the authors of [10], there are three basic strategies for routing the cooling liquid flow in conformal channels (which cover all of the other design concepts): zigzag, parallel, and spiral. Figure 2 shows the schematic flows of a cooling liquid, where the black lines depict the routing of the channels in a normal projection onto a cooled surface, and the red arrows show the directions of the cooling liquid flows.

In the “zigzag” strategy (also referred to as “serial”), the subsequent zones of a mould are being cooled one after another. In this case, the temperature of the cooling liquid continuously increases along the flow path. This results in the better cooling of the mould on the liquid inlet side yet worse cooling efficiency on the discharge side (Figure 2a). Serial cooling is generally not a preferred option unless the parts are small enough to neglect the delay. 

The construction of “parallel” cooling channels ensures the supply of a cooling liquid at the same temperature from a collective main line to a number of parallel cooling channels (Figure 2b). This solution has a disadvantage—any random increases in flow resistance that are caused by scale deposition result in the hampering of the cooling conditions in those sections. There is a positive feedback relationship between the slowing down of a flow and scale deposition; this leads to continuous increases in the differentiation of cooling efficiencies between parallel sections. The spiral type of a cooling channel (Figure 2c) is often applied in the manufacturing of parts that have curved or spherical shapes on the cooled surface. The cooling liquid flow in the channels can also be optimised by changing the profile and cross-section of the channel along its way [11]. This type of solution was applied in this study.

One of the most applied methods for designing and optimizing cooling channels in injection moulds is the so-called conformal line (or conformal surface) method [12,13]. This method can also be applied to designing cooling systems for pressure-casting moulds. The design of a conformal layout begins with the identification of conformal lines or surfaces that pass along the surface of a geometric model of a cast part or mould cavity. Then, based on these, the continuous routing of cooling channels for this form is designed. In the cases of parts with complex geometries, a practical way is to divide the mould cavity surface into less complicated sections and design individual cooling channel systems for each of these [14]. A systematic review of algorithm, designing, and optimization methods for cooling channels is provided in [15]. The number of publications that are directly related to the design of conformal cooling channels in die-cast moulds is significantly lower than those that cover injection moulds. 

In those publications that cover pressure-casting moulds, the authors have confirmed improvements in the cooling efficiency and the better uniformity of heat evacuations from moulds that are found in conformal cooling systems when compared to the traditional channel layout [6,16,17,18]. Reducing the average temperature in a die-cast mould that is cooled with a system of conformal channels ensures lower temperature gradients on the mould’s surface during the spray phase. Thanks to this, the level of strain in the area of the mould-forming surface is reduced, and its fatigue strength is increased [19,20,21]. Applying SLM printing technologies allows one to implement significant alterations to the construction of a mould cooling system. In such cases, the cooling channels may be located closer to the mould cavity’s surface. The spacing of the cooling channels can also be reduced significantly. The routing of the cooling channel’s axis can also be adapted to follow the shape of a cooled surface. When designing a cooling system for a mould or core to be manufactured by using the SLM technology, one should consider the mould cooling efficiency on one hand and its fatigue strength on the other [22]. In addition, minimising the number of operations of the cast component post-processing should be a priority.

This paper compares the microstructure and strength parameters of components that were cast in a mould using cores that were made in different ways. The properties of a series of castings that were produced in a mould with a traditional core made by machining from H13 steel (Set A of samples) were compared with the characteristics of those castings for which a maraging steel core with conformal cooling, printed by SLM, was used (Set B of samples).

## 2. Materials and Methods

HPDC die-forming elements are exposed to liquid casting alloy at high temperatures and pressures. Therefore, cores and mould inserts that have been manufactured using the SLM method are most frequently made of powders with chemical compositions that correspond to maraging tool steels that are dedicated to high-temperature working environments—DIN X3NiCoMoTi18-9-5. The manufacture of parts via the SLM method that includes such steel powders have adequate strength, plasticity, and wear strength when the heat treatment includes ageing. Those issues that are related to the strength of DIN X3NiCoMoTi18-9-5 steel powders, thermal processing parameters, and microstructure have been the subject of a number of studies and publications [23,24,25,26,27,28,29,30,31]. Applying a conformal mould or a mould core-cooling system allows for much better control of the alloy crystallization and cast solidification processes when compared to traditional solutions [32]; in this way, it is possible to have control over the microstructure, porosity, and strength properties of a cast element.

To determine the impacts of traditional and conformal cooling cores on the shape-dependent strength and microstructure of a casting alloy, a series of casts were produced in a FRECH cold-chamber HPDC machine. The casts were made of the Al Si9Cu3FeZn alloy (ISO 3522:2007). The chemical composition of the alloy (Table 1) was determined by the SPECTROMAXx LMF07 Stationary Metal Analyser (Kleve, Germany). The alloy casting temperature was 690 °C.

**Figure 3:**
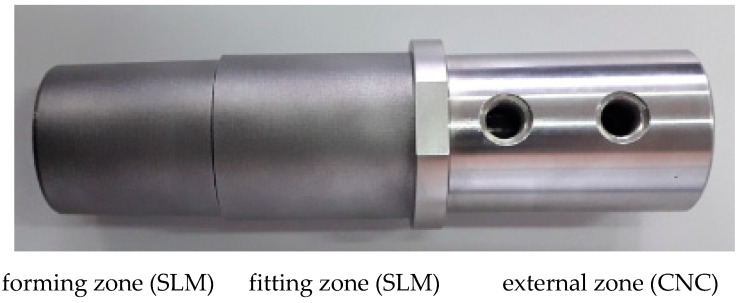
Forming core installed in a fixed section of pressure mould—hybrid construction.

This study aimed to compare the strengths, porosity, and microstructures of HPDC casts that were made in moulds that were alternatively equipped with traditional and conformal cooling cores. Cores with the traditional cooling core were manufactured with machined H13 steel. The conformal cooling cores were created by the SLM additive method from X3NiCoMoTi18-9-5 steel powder (commercial designation—MS1). These cores were made using the EOS M 290 device. The material was printed in an argon atmosphere with the following parameters: laser power—380 W; laser speed—1500 mm /s; laser beam diameter—80 μm; hatch space—90 μm; layer thickness—40 μm. To reduce costs, the working parts of the conformal cores (which consisted of the forming and fitting zones—Figure 3) were SLM-printed on the external section (which was made using traditional machining technology). The cores were aged at 490 °C for eight hours.

The thermo-physical properties of the mould and casting materials were taken from the ProCAST/Visual-Environment 15.0 database. It was assumed that the elements of the pressure mould were made of H13 steel and the casts were made of the AlSi9Cu3FeZn alloy. In the case of conformal cooling cores that were manufactured using the SLM method, 18Ni300 steel properties were assumed.

**Figure 4:**
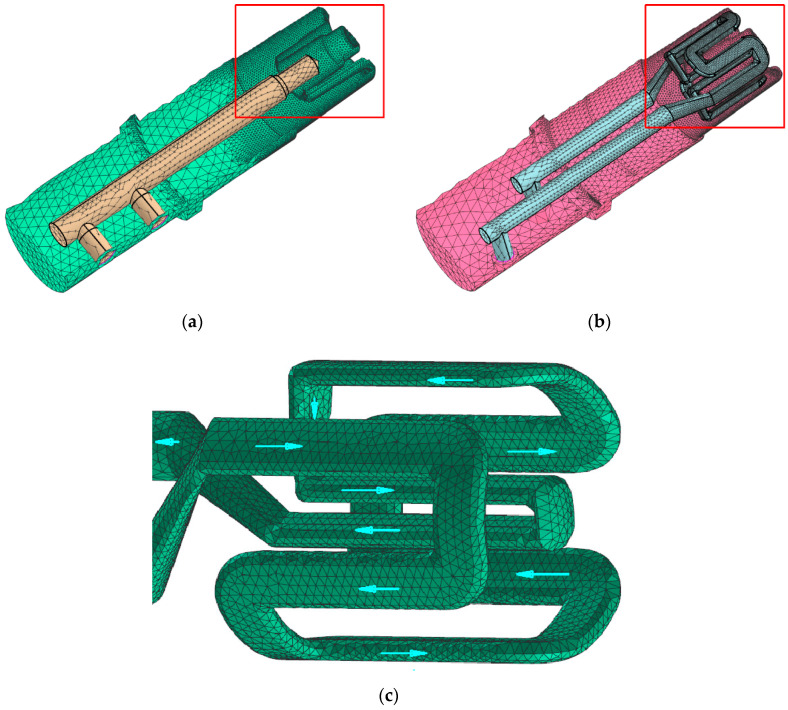
Shapes and positions of cooling system channels in cores that were made using the following methods: machining (**a**); machining + SLM (**b**); detail of conformal channel routing with indications of cooling liquid’s flow direction (ProCAST) (**c**).

The key difference between the two core designs was the shape and position of each thermal stabilization channel. A comparison of the traditional and conformal cooling-core layouts is shown in Figure 4.

**Figure 5:**
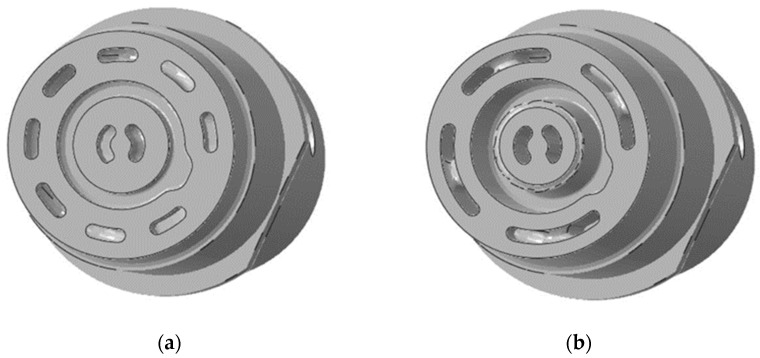
Cross-sections of a conformal core that was manufactured using SLM printing method: shape of cooling channels in the bottom section of core (located closer to machined part of core) (**a**); the shape of channels in the apical section of core (**b**).

In the part of each core that has no common contact surface with the cast, the cooling channels share the same geometry in each type of core. When recreating the shape of a cast, the conformal channels cool both the inner and outer surfaces of the cast in the forming section of the core. Figure 5 shows two cross-sections of a conformal cooling core that illustrate the positions and shapes of the channels.

**Figure 6:**
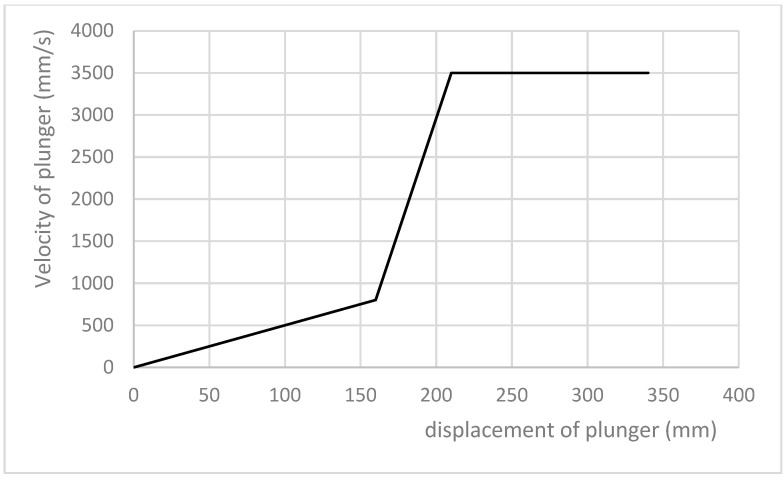
Plunger speed vs. position in pressing chamber.

Industrial casting trials were carried out using a FRECH 510 horizontal cold-chamber HPDC machine. The metal-pressing phase pressure was 172 bar. The mould was filled with the alloy in an 800-ms cycle with variable plunger speed (as shown in Figure 6).

During the industrial trials, the cycle time of the HPDC machine was 53 s, and the initial core-cooling water temperature was 40 °C. After being removed from the mould, the casts were air-cooled at 20 °C for approximately 20 min and then soaked in a water bath. The casts were analysed 20 days after their removals from the mould.

**Figure 7:**
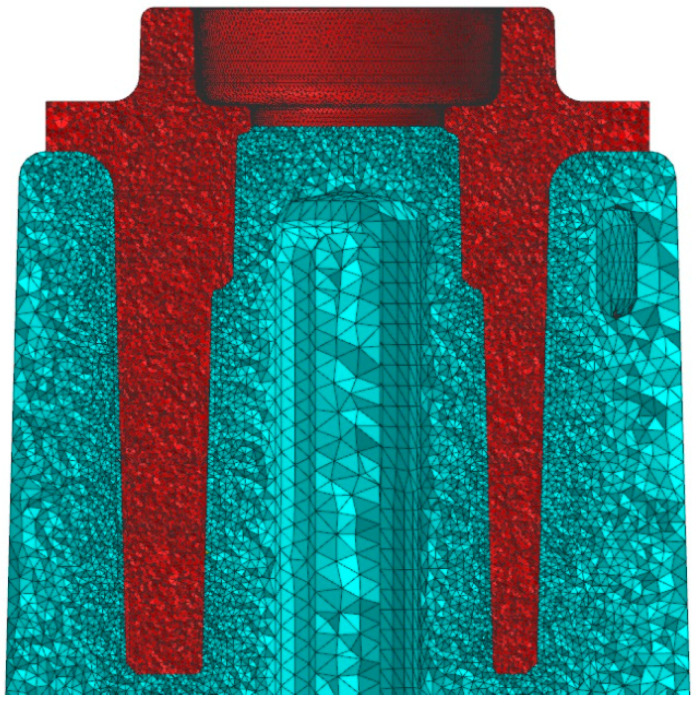
Cross-section of the fragment of FEM mesh of analysed core (aquamarine) and central zone of cast (red) in area of thermal and mechanical contact with core (ProCAST).

The shape-dependent strength measurements of the casts were carried out using an MTS 810 strength-testing machine. The test consisted of a compression of each cast following the stress diagram, which was previously determined based on finite element method (FEM) calculations. During the test, the changes in the force that were applied to the case and plunger displacements of the testing machine were recorded. Analyses of the metallographic specimens and observations of the porosity in the impact zones of the cores were carried out using a NIKON SMZ 745T (Tokyo, Japan) stereoscopic optical microscope, a NIKON ECLIPSE (Tokyo, Japan) metallographic microscope with DsFi1 camera digital image analysis, and a HITACHI (Tokyo, Japan) S-3400N scanning electron microscope with an Energy Dispersive X-ray Spectroscopy module made by NORAN System SIX by Thermo Fisher Scientific, Thermo Electron Scientific Instruments LLC, Madison, WI, USA. The metallographic samples were etched with Meyer Mi7Al (a 25% water solution of nitric acid). The chemical composition was analysed with an ED-XRF spectroscope using a SPECTRO MIDEX spectrometer (Kleve, Germany). The testing materials consisted of samples from the cast areas that were in contact with the core’s surface (Figure 7).

Evaluations of the alloy density in the casts were carried out by hydrostatic weighing. The relative density (ρ*_r_*) of the fabricated specimens is expressed by the following formula:

(1) $$\rho_{r}=\frac{m_{0}}{m_{0}-m_{1}}\rho_{1},$$

according to Archimedes’ principle, in which *m*_0_, *m*_1_, and ρ_1_ are the specimen’s weight in air, its weight when submerged in water, and the density of the applied water under measurement conditions, respectively.

The results of the experimental tests were compared to the numeric calculations that were carried out by using commercially available software such as ProCAST 14.5, ESI Group^®^, and Abaqus Dassault System Simulia (v. 2019).

**Figure 8:**
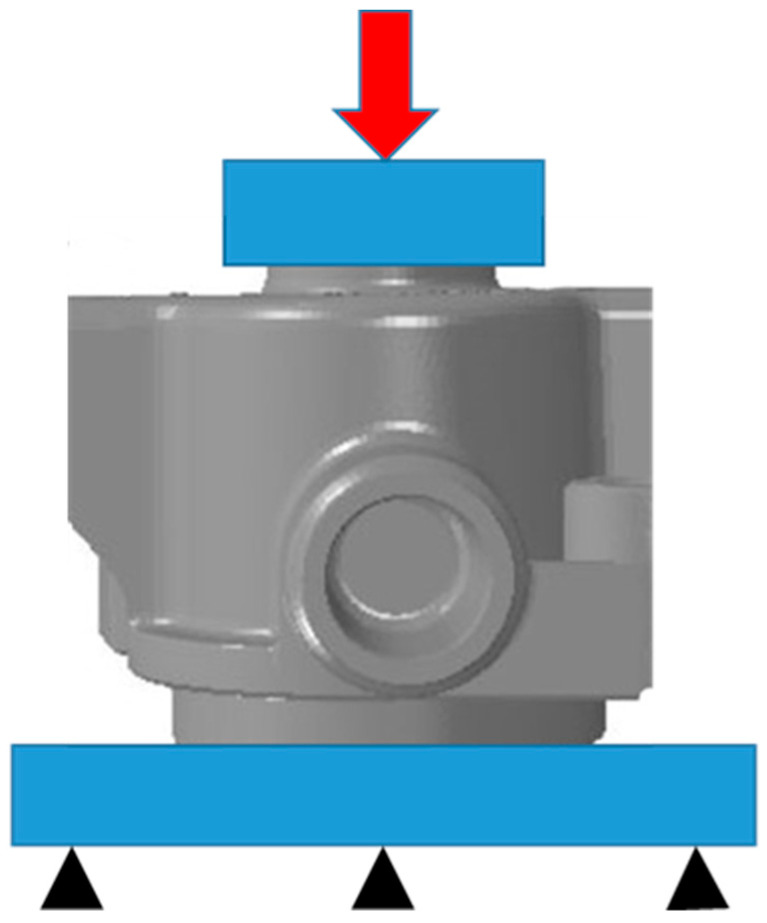
Schematic stress diagram for shape-dependent strength measurements that were carried out using MTS 810 strength-testing machine.

Due to the dimensions and shapes of the casts that were tested in the study, it was not possible to collect samples for stretching-strength tests. Therefore, compression tests were carried out using the MTS 810 strength-testing machine (following the stress scheme that is shown in Figure 8) to compare the shape-dependent strength of the casts in the traditional and conformal cooling-core impact zones. During the measurements (which were controlled by the displacement of the strength-testing machine plunger), all changes in force *F* were measured. The maximum recorded value (*F*_max_) was recognised as a measure of the shape-dependent strengths of the casts in the impact zones of the traditional and conformal cooling cores. 

## 3. Results

### 3.1. Strength Analysis

**Figure 9:**
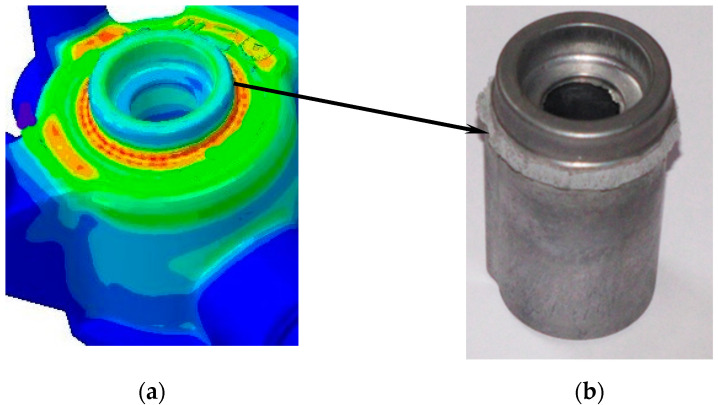
Zone of cast destruction caused by compressing force *F*_max_ that was applied following diagram in Figure 8: equivalent stress σ_o_ according to Mises hypothesis (**a**); photograph of the broken part (**b**).

The locations of the cast destruction zone that were caused by the impact of the compression forces that followed the stress diagram shown in Figure 9 were initially determined based on the FEM calculations. In the ring-shaped area shown in Figure 9a (in red), the equivalent stress σ_o_ that was determined by using the Mises hypothesis reached 260 MPa; this value is close to the alloy’s strength limit (270 MPa). The compressing force that was applied to the cast caused a break in its central part (as shown in the photograph in Figure 9b). The location and shape of this part of the cast corresponds to the section of the cast’s FEM mesh that was shown in Figure 7.

**Figure 10:**
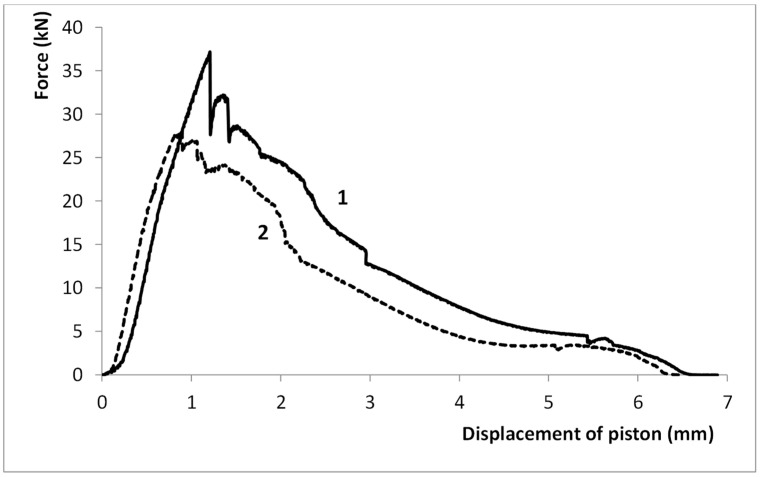
Typical graph of changing force that caused detachment of central part of cast located in thermal impact zone of mould core: (1) with conformal pattern of cooling channels, series B; (2) with traditional position of cooling channel, series A.

Twenty-eight casts were subjected to compression tests; fourteen each from Series A and Series B. In each of the performed tests, the shape and location of the core destruction zone were the same as the others (as shown in Figure 9). A significant difference was observed between the average value of the maximum destructive force in those casts that were made with conformal core moulds and those that were made in moulds with a traditional cooling-channel pattern. A typical graph that illustrates the changes in force *F* that caused the detachment of the central part of the cast (recorded during the compression tests) is shown in Figure 10. 

The good consistency of the maximum destructive force *F*_max_ that was registered in the conformal core casts could be found throughout the compression tests; however, the values of the destructive force in those casts that were made with a traditional core mould were more variable. For the traditional core-cooling moulds, the values of the destructive force were 28.83 ± 2.19 kN, while in the case of those casts that used conformal core moulds, the values were 37.01 ± 0.84 kN. This means an average increase of 28% in maximum destructive force *F*_max_ in the case of those elements that were cast in conformal core moulds as compared to the forces that were recorded in the tests of those casts that were produced with traditional cooling-core moulds.

The experimental results are shown below, as are the results of the pressure-casting simulation that was aimed at determining the impact of the core-cooling method on the microstructure of the alloy and cast porosity and indirectly on the shape-dependent strength of the casts that were made in the traditional and conformal core-cooling moulds.

### 3.2. Alloy Porosity Analysis

#### 3.2.1. Results of Porosity Modelling

Numerical simulations of the casting process ma were carried out using the ProCAST 14.5, ESIE Group^®^ software to identify the reasons for the shape-dependent strength in the casts that were made in moulds with the different types of core cooling.

Geometric models of the cast, moulds, and cores were prepared using the SolidWorks software and transferred in the STEP format to the ProCAST mesh generator. The finite element meshes for the geometric models of the inlet system, casts, and moulds were designed in the Visual-Mesh application (an integral component of the Visual Environment). The density of the FEM mesh was selected based on the wall thicknesses of the volumetric objects that were analysed. For almost the entire model of the cast, the assumed mesh size was 1.5 mm; however, this was reduced to 1 mm for the thinnest fragments (the injection slots and overflows). The mesh step in the inlet system was assumed to be 2 mm, and the thermal-stabilization channel ranged between 3 and 4.5 mm. The maximum mesh steps on the outer walls of the forming inserts that were distant from the surface of the socket were restricted to 10 mm. 

The material properties of the mould and cast were taken from the ProCAST/Visual-Environment 15.0 database. It was assumed that the elements of the pressure mould were made of H13 steel, and the casts were made of the AlSi9Cu3FeZn alloy. In the case of the conformal cooling cores that were manufactured using the SLM method, 18Ni300 steel’s properties were assumed. The assumed injection temperature and parameters of the HPDC machine were identical to those that are indicated in the “Materials and Methods” section of this paper. 

**Figure 11:**
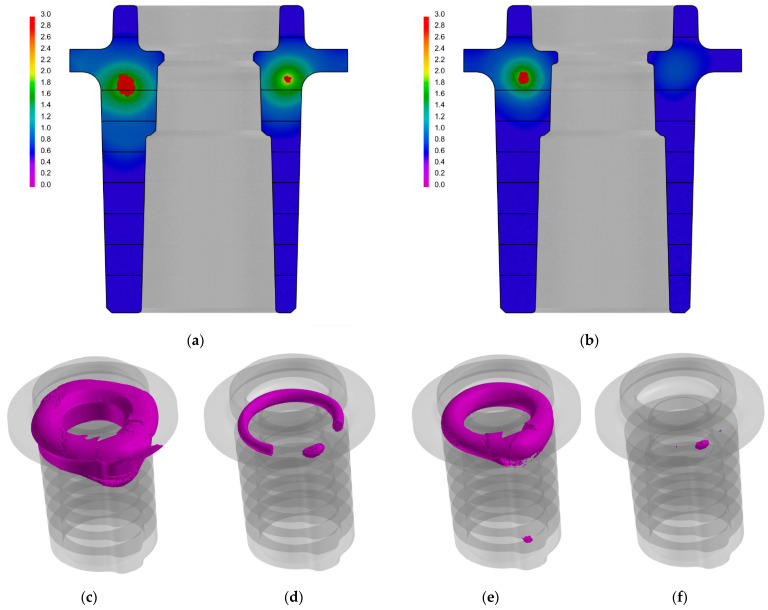
Comparison of areas of occurrence and volume fraction of porosity in central sections of casts that were manufactured in moulds with traditional core-cooling system (**a**) and conformal core-cooling system (**b**) and zones of macro- and micro-porosity (greater than 1% vol.: (**c**,**e**)) and macro-porosity (greater than 2% vol.: (**d**,**f**)) for traditional core-cooling system (**c**,**d**) and conformal one (**e**,**f**).

The results of the performed numerical calculations were used to determine any changes in temperature in the cast and mould and, furthermore, to determine the percentage share of the porosity in the cast walls. Comparisons of the areas of porosity occurrence and the volumetric share of the contraction defects in the central parts of the casts that were made in the traditional and conformal cooling cores are shown in Figure 11. It was assumed that the area of macro porosity was characterized by a volume fraction of cavities that was greater than 2%.

To allow for a more precise determination and comparison of the volumetric shares of porosity in the casts, the geometric models of their central sections were divided into nine horizontal zones of a nearly annular shape, and the porosity percentages were determined in these sections. The results are shown in Table 2.

**Figure 12:**
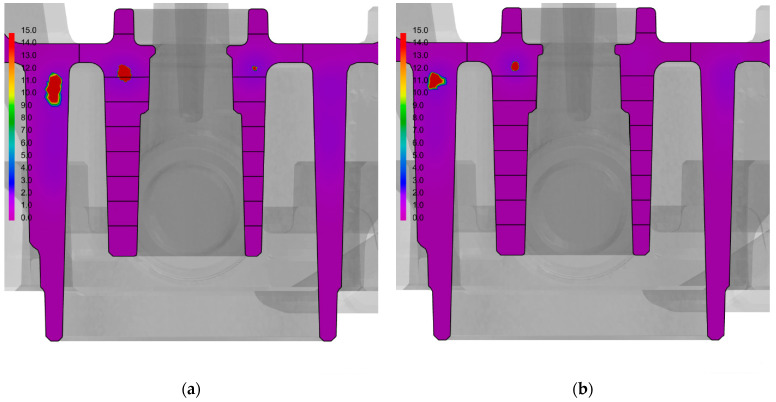
Comparison of areas and volume fractions of porosity in casts that were produced in traditional cooling-core moulds (**a**) and conformal cooling-core moulds (**b**).

Figure 12 shows a comparison of the occurrence and extent of the porosity areas in a cross-section of the entire cast. 

#### 3.2.2. Investigation of Alloy Porosity and Density

**Figure 13:**
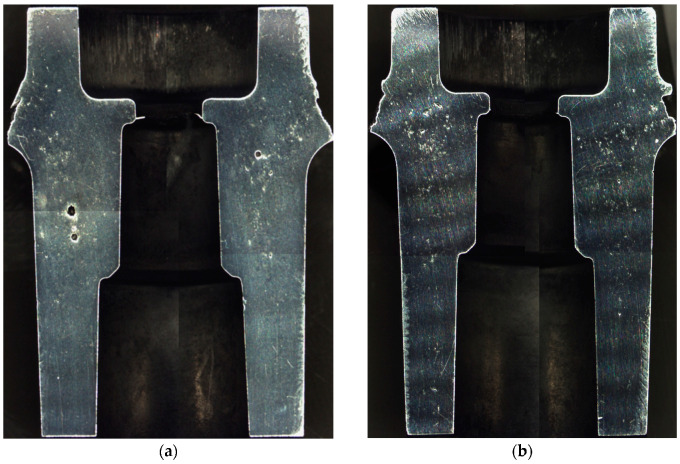
Porosity in cast wall section exposed to experimental core in casts made with moulds with traditional cores (**a**) and conformal cores (**b**).

Figure 13 shows an image of the test surface; this figure also shows the distributions and sizes of the shrinkage porosity that are typical for traditional (13a) and conformal (13b) cores. In the case of a traditional core, the porosity was more concentrated and occurred in the forms of large areas that were difficult to feed. Casting the element in a conformal core mould caused higher dispersion and reduced the percentage share of the contraction porosity in the casting zone that was impacted by the conformal cooling system.

Alloy density measurements were also performed using the cast parts that broke off during the compression testing (before cutting them for metallographic analysis). Twenty-eight samples (fourteen traditional mould casts, and fourteen conformal casts) were measured using the Archimedes method.

The results of the statistical analysis of the sample parameters are shown in Table 3. The boundaries of the confidence intervals were determined to be at a 95% confidence level. As demonstrated in the measurements, the alloy’s density in the conformal core impact zone was significantly higher than it was in the impact zone of the traditional core (with average values of 2.6796 g/cm^3^ and 2.6638 g/cm^3^, respectively). Significant differences also occurred in the cases of the destructive forces and the SDAS comparisons between the casts that were made with the traditional and conformal core-cooling technologies. The lower and upper limits of the confidence interval that are indicated in Table 3 were determined for a confidence level of 95% (assuming a normal statistical distribution of the analysed variables).

### 3.3. Microstructure Analysis

The analyses of the alloy’s microstructures that were performed in the study were aimed at comparing the sizes of the dendritic cells in the areas of impact in the experimental core-cooling systems. Microstructure analyses and evaluations of the alloy’s porosity were made on the surface of samples that were obtained by crosscutting the central parts of any casts that broke off along their central axes during the compression test.

#### 3.3.1. Results of Microstructure Modelling

The distinctive impact of the conformal cooling system on reducing the volume fraction of the porosity in the cast is visible in the second, third, and fourth layers that are identified in the central section of the cast. These layers encompass the area in which the central part of the cast was broken off during the tests. It is therefore likely that any reduction in the porosity share may result in increases in the shape-dependent strength of a cast in this area.

The second factor that may influence the shape-dependent strength of a cast element in the destruction zone may be its microstructure; this means the sizes and number of any intermetallic phase separations, the size of the dendrites in the Al-based solid phase, the SDAS (secondary dendrite arm space) value, and the features of the phase grain in the eutectic.

**Figure 14:**
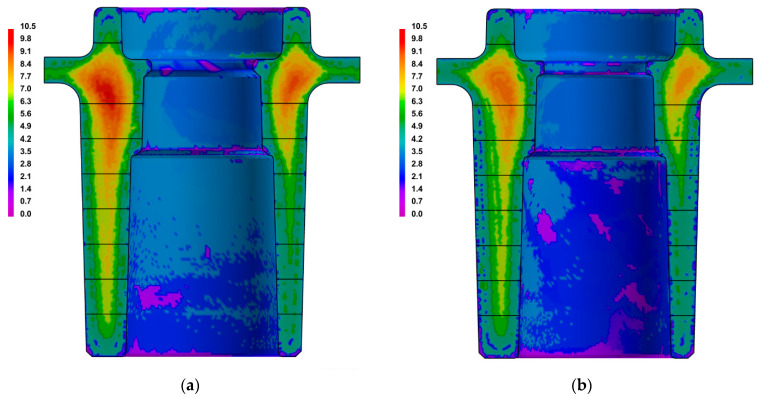
Comparison of SDAS parameters in central sections of casts that were made using moulds with traditional core-cooling layout (**a**) and conformal cooling-channel layout (ProCAST) (**b**).

The results of the numerical calculations were used to compare the SDAS dendrite arm spacing in the cooling-core impact zones; this was used as a measure of the alloy’s structure fragmentation. A comparison of the SDAS parameters in the central section of the casts that were made in traditional and conformal core moulds is shown in Figure 14.

**Figure 15:**
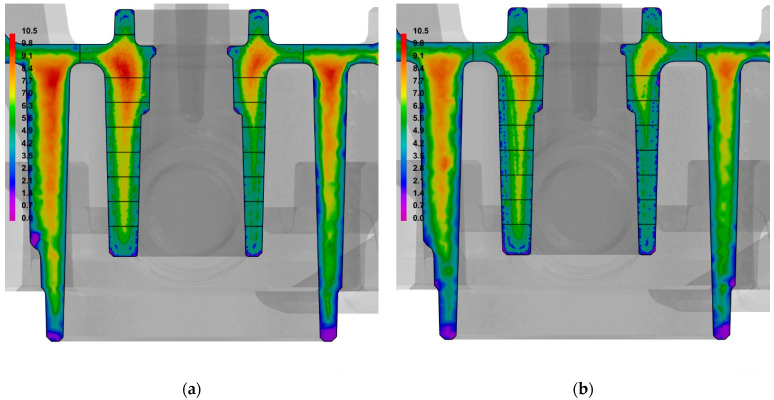
Comparison of SDAS parameters in casts that were made using moulds with traditional core-cooling layout (**a**) and with conformal cooling-channel layout (**b**).

Figure 15 shows a comparison of the SDAS parameters over an entire cross-section of the casts.

Table 4 reviews the determined SDAS values based on the numerical models of the casts and moulds (with both traditional and conformal core-cooling systems).

The noticeable impact of the conformal cooling system on the microstructure fragmentation is visible in all but the first zone (into which the model of the cast’s central section was divided) as well as in the outer walls of the cast. The fragmentation of the alloy’s microstructure that was indicated by a decrease in its SDAS value may also be a reason for the increased shape-dependent strength of the cast in the alloy’s de-cohesion zone (as was demonstrated in the compression tests).

#### 3.3.2. Microstructure Investigations

**Figure 16:**
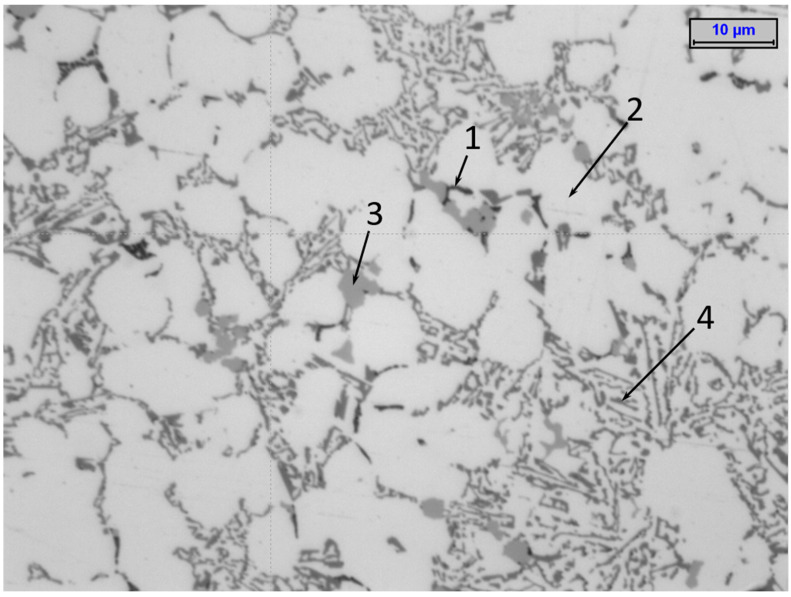
Typical microstructure of alloy in analysed samples of Al Si9Cu3FeZn that indicate presence of (1) Al_2_Cu, (2) α-Al dendrites, (3) primary phase Al_15_(Fe, Mn)_3_Si_2_, and (4) eutectic Al-Si-Al_2_Cu.

**Figure 17:**
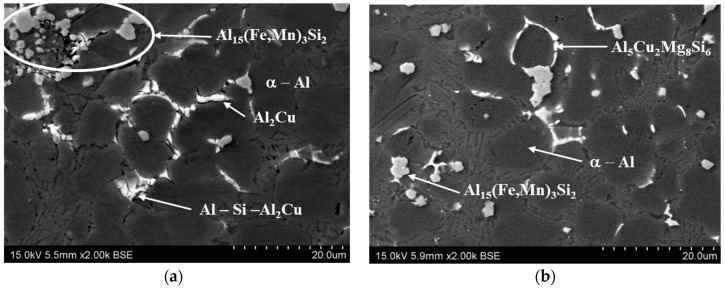
Distributions of phases that occur in microstructure of Al9Cu3(Fe)(Zn) alloy that were determined using scanning microscope; photographs indicate alloy’s reinforcing phases in Al_2_Cu (**a**) and Al_5_Cu_2_Mg_8_Si_6_ eutectic (**b**).

The Al Si9Cu3FeZn alloy’s microstructure indicated a degree of disorder that is typical for pressure casting (with its very high speeds of mould-filling with liquid metal and its very high values of applied pressure to the solidifying alloy) (Figure 16). The alloy contained typical phases such as α-Al dendrites with high aluminium contents, an Al-Si-Al_2_Cu eutectic, precipitates of the primary Al_15_(Fe, Mn)_3_Si_2_ phase, and (in some cases) intermetallic precipitates of the Al-Cu phase (which provided reinforcement of the alloy). The phases that were observed using an optical microscope were further identified with scanning microscopy. Figure 16 and Figure 17 show the grain distributions as well as the shapes of the basic phases and their identifications in the microstructure of the alloy.

The application of the conformal cooling cores accelerated the evacuation of the heat from this section of the cast; as a consequence, this led to a reduction in the α-Al dendrite grain cross-section dimensions as compared to their sizes in the slower-cooling alloy in the traditional core mould setup.

**Figure 18:**
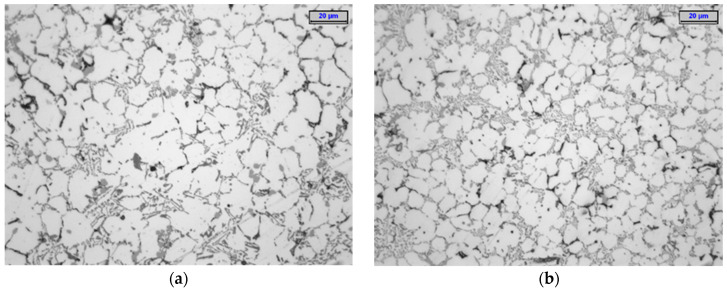
Microstructure of pressure-cast AlSi8Cu3(Fe)(Zn) alloy: traditional core mould (**a**); conformal core mould (**b**).

This difference is discernible in the images of the metallographic samples that were cut from the casts that were obtained from the traditional and conformal core moulds (as shown in Figure 18).

Besides the quality assessment of the α-Al-phase grain size, the SDAS parameter values were also measured. The values of the SDAS also changed in the function of each cast’s wall thickness. Despite the above conditions, the minimum and maximum values were determined in both the entire cross-section of the analysed fragment of the cast that is shown in Figure 13 and (using the shape-dependent strength measurements) in the breaking zone from the central part of the cast element; i.e., Zone 2 (Table 3). The minimum measured SDAS value for the entire cross-section of the cast was approximately 6 μm for the traditional core mould cast (a maximum value of 13 μm, and an average value of 9.1 μm for 30 measurements). In Zone 2, the maximum measured value of the SDAS was approximately 7 μm. The minimum measured SDAS value in the case of the casts from the conformal core moulds was approximately 3 μm (with a maximum value of approximately 12 μm). The average value of the 30 measurements was 8.2 μm in this case. In Zone 2 of the conformal core mould cast cross-section, the maximum measured SDAS value was approximately 6 μm. The results of the SDAS measurements are reviewed in Table 3.

## 4. Discussion of Results

The application of conformal cooling channels in an SLM core increased the rate of heat evacuation from the cast when compared to the traditional cooling setup. This effect was obtained thanks to an increase in the surface area of the channels in the part of the core that was exposed to the metal as well as to the adaptation of their geometry to the surface of the “cast element-core” interface (Figure 4 and Figure 5). In addition, the CAD geometric model indicated that the channel volume of the conformal system increased by nearly two-fold as compared to the volume of the traditional linear channel that was used in the traditional setup. The surface area of the cooling channels increased five-fold. To reduce the cost of the cores, the design was based on a hybrid layout with the conformal sections printed over the CNC element with straight linear channels. The CNC and SLM parts were made of the same grade of maraging-type steel.

The increase in the rates of the heat transfer from the cast influenced the alloy’s microstructure and decreased the porosity in the cast; this increased the shape-dependent strengths of the parts. Due to the shape of the cast, extracting standard alloy strength-testing samples was not possible. Moreover, the objective of the study was to analyse the impact that applying conformal and traditional cooling systems had on the alloy’s properties in the direct impact zones. Therefore, a decision was made to carry out the shape-dependent strength tests by stressing the alloy in such a way as to focus the de-cohesion of the material in the zones of the direct impacts of the traditional and conformal cores. The experimental tests were preceded by a numerical load simulation of the cast. The results of this simulation allowed for a precise determination of the destruction zone and determination of the stress values during the compression test of the casting (Figure 9a). The assumed stress load-distribution scheme shown in Figure 8 resulted in a breakage of the central part of the cast by the compression force that was induced by the movement of the strength-testing machine plunger. The maximum value of the compressive force recorded during the tests was taken as the value of the shape-dependent strength of the castings in the cooling zones of both types of cores (Figure 10)

The research was carried out on 28 castings, 14 each from series A and series B. In the case of the traditional core application, the average value of maximum destructive force *F_m_* was 28.83 kN; in the case of the conformal cores, this force was greater (reaching 37.01 kN). This translates to an increase of approximately 28%. It is worth noting that the values of the destructive force in the casts from the conformal core moulds demonstrated smaller variations than those that were recorded in the cases of the traditional cooling-core casts. An analysis of the distributions and shapes of the contraction porosities in the de-cohesion zones in the walls of the cast fragments that broke off during the strength tests of the traditional cooling casts indicated the potential occurrence of rather large cavities; this may significantly reduce the cast’s strength. This phenomenon did not occur in the moulds with the conformal core-cooling system; in these cases, the sizes of the individual cavities were much smaller.

The alloy densities that were determined using the hydrostatic weighing (Archimedes) method in the central (broken-off) fragments of the casts from the traditional core moulds was 2.6646 g/cm^3^; this was 2.6791 g/cm^3^ in the cases of the casts from the conformal core mould. This translates to an increase of approximately 0.55% when using conformal cooling moulds. The experimental measurements were complemented with a numerical simulation that was aimed at identifying the locations and making quantitative evaluations of the shrinkage porosity in the castings. The shrinkage porosity in Zone 2 (Table 2) had a smaller range of occurrence in the cases of the casts from the conformal core mould. In addition, its average value (0.766% in Zone 2 for conformal cooling) was lower than in the cases of the traditional cooling casts with a porosity of 1.218% in this zone.

Such a significant difference in the average values of the destructive force in the casts from the moulds with the two different cooling systems indicates the strong impact of the conformal cooling-channel system on the alloy-solidification process. The analysis of the alloy’s microstructure demonstrated that the increase in the casting strength did not result solely from the reduced contraction porosity in the alloy. The microstructure of the Al Si9Cu3FeZn alloy demonstrated a high degree of disorder, which is typical for pressure-casting objects (this is caused by the very high speed of the mould filling with liquid metal). The typical phases were identified in the alloy, such as α-Al dendrites with high aluminium contents, the Al-Si-Al_2_Cu eutectic, precipitates of the primary Al_15_(Fe, Mn)_3_Si_2_ phase, and (in some cases) the Al_2_Cu and Al_5_Cu_2_Mg_8_Si_6_ phases, the last two phases contributing to reinforcing the alloy’s structure [33]. 

Due to the natural alloy ageing process that was applied in this study, all the tests were carried out 15 days after the casting (after the mechanical properties of the alloy stabilized) [33]. The application of the conformal cooling system resulted in the finer grain structure of the alloy as compared to the traditional cooling setup. This is visible in Figure 18, which compares the alloy’s microstructures in the castings made with the traditional (Figure 18a) and conformal (Figure 18b) cores. An SDAS measurement was performed in addition to the quality analysis; the values of this parameter in the analysed cross-section of the cast (Figure 13) that was made using the traditional core mould ranged from 6 to 13 μm, while the maximum value of SDAS was 7 μm in Zone 2. The SDAS parameter that was calculated in the numerical simulation of this zone was approximately 7 μm. The SDAS value in the cross-section of the cast that was made using the conformal core mould ranged between 3 and 12 μm. This measurement narrowed down to Zone 2—ultimately yielding a maximum SDAS value of approximately 6 μm. The numerical simulation estimated the SDAS value in Zone 2 to be 6.3 μm.

The results of the measurements indicated that the SDAS parameter value depends on the cooling rate of the cast; this means that it directly depends on the applied type of cooling system and the thickness of the cast wall. Figure 14 and Figure 15, and Table 4 show the results of the numerical simulations of the SDAS values in the cross-sections of the casts. The analysis of the impacts of the conformal core-cooling system on the alloy’s microstructure demonstrated that the structure fragmentation that was measured with the SDAS parameter occurred in the entire impact zone, while the decrease in the contraction porosity percentage (as compared to the traditional core-cooling system) was only discernible around the hot spots that occurred in the cast within the core thermal impact range. The experimental and ProCAST simulation results were found to be consistent.

## 5. Conclusions

The research was aimed at determining the influence of the use of a conformal cooling system of a mould on the increase in strength of a pressure casting as compared to castings that were obtained in a mould with a traditional cooling system. Such a comparison is limited to the area of the casting that was directly affected by a conformal cooling system. As a result of these studies, information was obtained about the destructive force of the casting in the zone of influence of the conformal cooling channel. The value of this force can be compared with the destructive force of the same casting in the same area that was made in a cooled mould traditionally.

The laboratory and industrial analyses that were performed in this study (further complemented with numerical calculations) demonstrated that the locations of the cooling system’s channels have a quite significant influence on the properties of the alloy as well as the strength of the element that was cast in a pressure mould. The industrial tests consisted of two series of casts in the same mould. In the first series, the mould was fitted with traditional cooling cores and linear cooling channels. In the second series, these were replaced by cores with a conformal layout of the cooling channels. Several hundred casts were produced and subjected to strength tests that were aimed at determining the maximum force that led to the destruction of the cast element. The tests demonstrated that the destructive force in the case of the conformal core mould casts was 28% higher than in the case of the maximum destructive force that was measured in the traditional core mould casts (a linear layout of the cooling channels). The alloy’s de-cohesion plane was located in the effective zones of both types of cores. The reason for such an increase in the casting strength was the decrease in the percentage of the shrinkage porosity on the one hand and the changes in the microstructure of the solidifying alloy on the other. In the case of the casts that were produced using a conformal cooling core, no individual large cavities were found (as opposed to the traditionally cooled cast elements).

The increase in the cooling rate that was made possible by applying the conformal cooling cores had a direct impact on the size reduction of the dendritic grains during the alloy-solidification process. The average value of the SDAS parameter that was determined in the cross-section of the cast wall was reduced by approximately 11% in the area of the conformal core impact as compared to the values that were measured in the cast elements from the traditional cooling-core mould. Optimizing the core-cooling process was possible by applying hybrid design cores that combined CNC and SLM technologies. The conformal core-cooling system presents an opportunity to affect the structure of the cast alloy by changing the intensity of the cooling by controlling the cooling liquid’s temperature.

The aim of the presented research was to verify the applicability of pressure mould components that are manufactured using the SLM method for the high-volume production of cast components with improved quality. On the other hand, future research directions, on the other hand, will include an analysis of the influence of the conformal cooling system on the fatigue strength and durability of pressure mould components that are produced by the SLM method.

## Figures and Tables

**Table 1:** Chemical composition of the alloy used for tests in wt.% (Al—base).

Si	Fe	Cu	Mn	Mg	Cr	Ni	Zn	Pb	Sn	Ti
9.31	0.79	2.1	0.30	0.35	0.04	0.53	0.91	0.08	0.03	0.03

**Table 2:** Volume fraction of porosity in casts made with traditional and conformal cores (simulation results).

Number and Position of the Layer		Porosity in Cast (%)	
		Traditional Channel	Conformal Channel
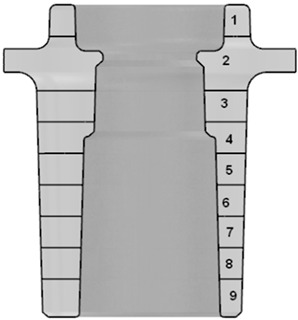	1	0.574	0.570
	2	1.218	0.766
	3	1.281	0.657
	4	0.728	0.571
	5	0.581	0.573
	6	0.572	0.575
	7	0.573	0.576
	8	0.574	0.577
	9	0.575	0.577

**Table 3:** Average values and confidence intervals for alloy characteristics in core thermal impact zones.

Parameter	Unit	Traditional Core			Conformal Core		
		Average	Lower Limit	Upper Limit	Average	Lower Limit	Upper Limit
Destructive force	kN	28.8	27.2	30.0	37.0	36.4	37.6
SDAS	μm	9.1	7.2	11.0	8.2	5.4	11.0
Density	g/cm^3^	2.6638	2.6606	2.6670	2.6796	2.6745	2.6847
Density deviation *	g/cm^3^	0.0879	0.0911	0.0847	0.0721	0.0772	0.067

* Deviation from the theoretical alloy density level 2.7517 g/cm^3^ at ambient temperature.

**Table 4:** Average values of determined SDAS parameters based on calculations that were performed with ProCAST software in cases of casts made with traditional and conformal core moulds.

Number and Position of Layer		SDAS (μm)	
		Traditional Channel	Conformal Channel
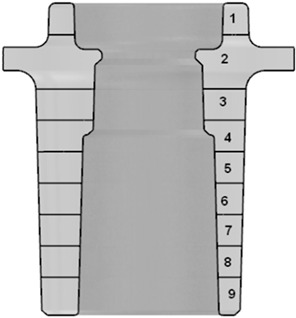	1	4.68	4.64
	2	6.79	6.28
	3	6.88	6.11
	4	6.12	5.45
	5	5.64	5.01
	6	5.46	4.82
	7	5.25	4.75
	8	5.10	4.67
	9	4.48	4.02

## Data Availability

The data that support the findings of this study are available from the corresponding authors, [J.P.; A.B.; A.G.-K.], upon reasonable request.
